# Aberrant Intra- and Internetwork Functional Connectivity in Depressed Parkinson’s Disease

**DOI:** 10.1038/s41598-017-02127-y

**Published:** 2017-05-31

**Authors:** Luqing Wei, Xiao Hu, Yajing Zhu, Yonggui Yuan, Weiguo Liu, Hong Chen

**Affiliations:** 1 Key laboratory of Personality and Cognition, Faculty of Psychology, Southwest University, Chongqing, 400715 P.R. China; 2 Department of Neurology, Affiliated Brain Hospital of Nanjing Medical University, Nanjing, 210029 P.R. China; 3 Department of Psychiatry and Psychosomatics, Affiliated ZhongDa Hospital of Southeast University, Institute of Neuropsychiatry of Southeast University, Nanjing, 210009 P.R. China

## Abstract

Much is known concerning the underlying mechanisms of Parkinson’s disease (PD) with depression, but our understanding of this disease at the neural-system level remains incomplete. This study used resting-state functional MRI (rs-fMRI) and independent component analysis (ICA) to investigate intrinsic functional connectivity (FC) within and between large-scale neural networks in 20 depressed PD (dPD) patients, 35 non-depressed PD (ndPD) patients, and 34 healthy controls (HC). To alleviate the influence caused by ICA model order selection, this work reported results from analyses at 2 levels (low and high model order). Within these two analyses, similar results were obtained: 1) dPD and ndPD patients relative to HC had reduced FC in basal ganglia network (BGN); 2) dPD compared with ndPD patients exhibited increased FC in left frontoparietal network (LFPN) and salience network (SN), and decreased FC in default-mode network (DMN); 3) dPD patients compared to HC showed increased FC between DMN and LFPN. Additionally, connectivity anomalies in the DMN, LFPN and SN correlated with the depression severity in patients with PD. Our findings confirm the involvement of BGN, DMN, LFPN and SN in depression in PD, facilitating the development of more detailed and integrative neural models of PD with depression.

## Introduction

Depression is one of the most common non-motor symptoms of Parkinson disease (PD), with a prevalence of around 35%^1^ and an increasing incidence with progression of the disease^2^. Converging evidence indicates that depression in PD may be a consequence of the neurodegenerative process of the disease rather than a reactive process to the chronic, disabling symptoms^3^. Depression associated with reduced functioning and cognitive impairment is a key determinant of poor health-related quality of life in patients with PD^1, 4^. Understanding depression in patients with PD is, therefore, crucial to achieve the optimal diagnosis and treatment that is needed for patients with this disease.

Functional neuroimaging investigations of depression in PD can advance both the diagnosis biomarkers and treatment evaluation of this debilitating illness. With positron emission tomography (PET), single-photon emission computed tomography (SPECT), and task-based functional magnetic resonance imaging (fMRI), functional anomalies in several brain regions are related to depressed PD patients (dPD), including the dorsolateral prefrontal cortex (DLPFC), medial prefrontal cortex (MPFC), orbitofrontal cortex (OFC), anterior cingulate cortex (ACC), insula, thalamus, amygdala, ventral striatum, and caudate^5–9^. Those findings lend support to the viewpoint that prefrontal cortex, basal ganglia (BG), and limbic system are involved in dPD. More recently, resting-state fMRI (rs-fMRI), as a novel non-invasive approach to measuring baseline brain activity and connectivity, has been increasingly utilized to uncover the neural underpinnings of dPD^10–14^. Those rs-fMRI studies using the amplitude of low-frequency fluctuation (ALFF) and regional homogeneity (ReHo) methods highlighted that dPD patients had abnormal resting brain activity in the prefrontal and limbic regions, such as amygdala, OFC, DLPFC, MPFC, ACC, compared with non-depressed PD (ndPD) patients^10, 12–14^. Also reported were aberrant resting brain connectivity between regions of OFC-insula, OFC-amygdala, middle temporal gyrus (MTG)-putamen, amygdala-putamen, median cingulate cortex (MCC)-MPFC, and MCC-PCC/precuneus (PCC/PCu)^10–14^, suggesting disrupted functional integrity in prefrontal, cingulated, BG, and limbic areas in dPD patients. Overall, the above neuroimaging findings allow us to propose that depression in PD could depend on the damage to specific neural networks rather than on the dysfunction of single, discrete brain region. Attempting to understand dPD from a network-level perspective, hence, may yield an incremental advancement to existing neural models of dPD. Although former researchers using region-of-interest (ROI) approach have noted the potential benefits of exploring dPD at the neural-system level^10–14^, neural network disruption in dPD remains largely obscure.

Independent component analysis (ICA), as a powerful data-driven approach with no a priori definition of seed regions, offers an effective means for identification of functional systems within the brain during rest, typically referred to as “resting state networks” (RSNs) or “intrinsic connectivity networks” (ICNs)^15, 16^. The study of RSNs or ICNs has already been shown to be of great potential clinical value, providing rich and sensitive markers of PD^17–19^. To our knowledge, no study so far has investigated it in dPD. Given that several prefrontal, cingulated, BG, and limbic regions are well documented to be implicated in dPD^6, 9, 12, 13^, this study sought to determine (1) whether the corresponding neural networks composed of these regions displayed aberrant interactions within each network and between them (intra- and internetwork connectivity) in dPD patients by comparing with ndPD and healthy subjects and (2) if so, whether the detected aberrant interactions between dPD and ndPD patients were related to the severity of depression in PD. To address the aforementioned issues, ICA method was performed to isolate the ICNs comprising of BG network (BGN), default-mode network (DMN), salience network (SN) and frontoparietal network (FPN), which cover large parts of the prefrontal, cingulated, BG, and limbic areas relevant to dPD^6, 9, 12, 13^. In consideration of ICA model order selection having a significant effect on ICN’s characteristics^20^, this study acquired ICNs at 2 decomposition levels. The first is a relative lower ICA decomposition estimated using minimum description length criterion^21^, and the second is a higher ICA decomposition that has been applied in previous studies^20, 22^. At each decomposition level, functional connectivity within each individual network was evaluated using the corresponding ICN’s spatial z-maps. Interactions among networks were measured by Pearson’s correlation between ICN’s time courses^23^. Relationship between abnormalities of intra- and internetwork connectivity and clinical severity in patients with PD was assessed by Spearman correlation analysis. The results of this study will contribute to our knowledge of the neural network disruption in PD with depression.

## Results

### Demographic and clinical characteristics

Demographic and clinical features of the sample were listed in Table 1. Age, gender, education level, and MMSE score were not significantly different among the three groups. No significant difference in disease duration, Hoehn and Yahr (H&Y) stage^24^, the motor component of the Unified Parkinson’s Disease Rating Scale (UPDRS-III)^25^ score, and levodopa equivalent dose (LED) were found between dPD and ndPD patients. By definition, the Hamilton Depression Rating Scale (HDRS) scores of dPD patients were significantly higher than that of ndPD patients (p < 0.001).

**Table 1:** Demographic and clinical characteristics of the total sample.

Groups	HC(N = 34)	ndPD(N = 35)	dPD(N = 20)	P Value
Age (years)	57.26 ± 5.95	57.80 ± 7.11	58.30 ± 7.66	0.86*
Education (years)	11.62 ± 4.91	10.69 ± 3.29	11.15 ± 3.12	0.62*
Gender (male:/female)	16/18	19/16	8/12	0.58***
HDRS	1.91 ± 2.48	7.06 ± 3.11	19.80 ± 4.37	< 0.001*
MMSE	29.12 ± 1.77	28.66 ± 1.66	28.60 ± 1.10	0.39*
UPDRS-III	—	27.24 ± 13.39	28.95 ± 13.14	0.65**
H&Y	—	1.77 $\pm$ 0.68	1.45 $\pm$ 0.58	0.07****
Disease duration	—	6.06 ± 3.53	5.45 ± 2.84	0.51**
LED (day/mg)	—	474.0 ± 375.67	512.8 ± 361.07	0.71**

*Comparisons of Age, Education, HDRS and MMSE among three groups used one-way ANOVA; **Differences of HARS, UPDRS-III, Disease duration and LED between ndPD and dPD calculated using two-sample t test; ***Gender distribution in three groups assessed by chi-squared test; ****Comparison of H&Y between ndPD and dPD utilized Wilcoxon rank sum test. Abbreviations: HC, healthy control; ndPD, non-depressed Parkinson’s disease; dPD, depressed Parkinson’s disease; HDRS, Hamilton Depression Rating Scale; MMSE, Mini-Mental State Exam; UPDRS, Unified Parkinson’s Disease Rating Scale; H&Y, Hoehn&Yahr staging; LED, L-dopa equivalent daily dose.

### Intranetwork connectivity analysis

**Figure 1:**
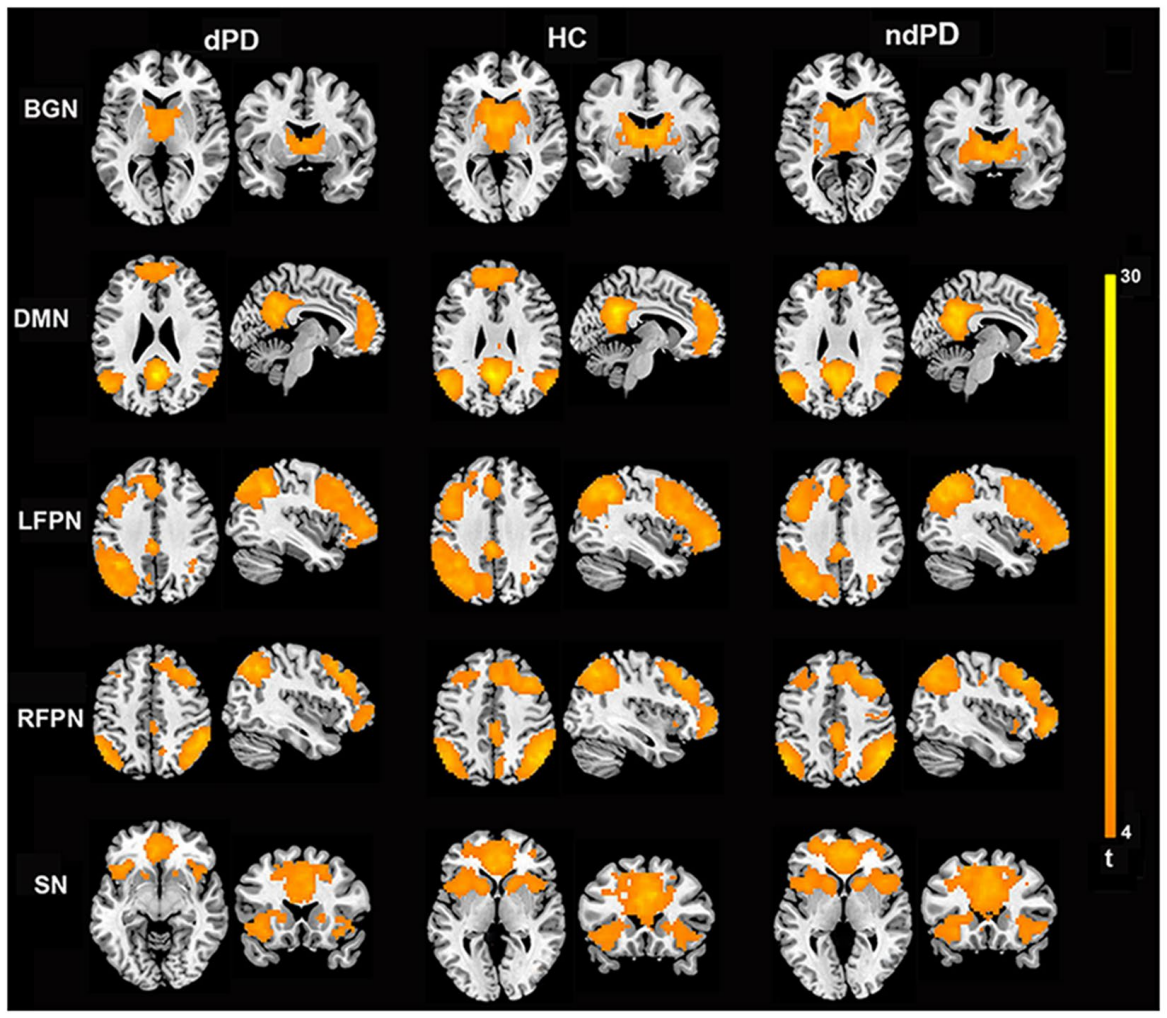
Spatial maps of BGN, DMN, LFPN, RFPN, and SN in dPD, HC, and ndPD group (one-sample t-test, p < 0.001, FDR corrected). The first two columns represented brain maps for dPD, middle two columns represented brain maps for HC, and last two column represented brain maps for ndPD.

At the 28-component level, the spatial maps of the 5 selected ICNs for each group are shown in Fig. 1 (one-sample t-test, p < 0.001, FDR corrected). Our procedure for independent component classification produced consistent ICNs^17, 22, 26^.

BGN: putamen, caudate, pallidum, and thalamus.

DMN: MPFC, PCC/PCu, medial and lateral temporal cortex, lateral parietal cortex (LPC).

Right/Left FPN (RFPN/LFPN): right/left posterior parietal cortex (PPC), and right/left DLPFC.

SN: anterior insula, ACC, and several subcortical and limbic structures.

**Figure 2:**
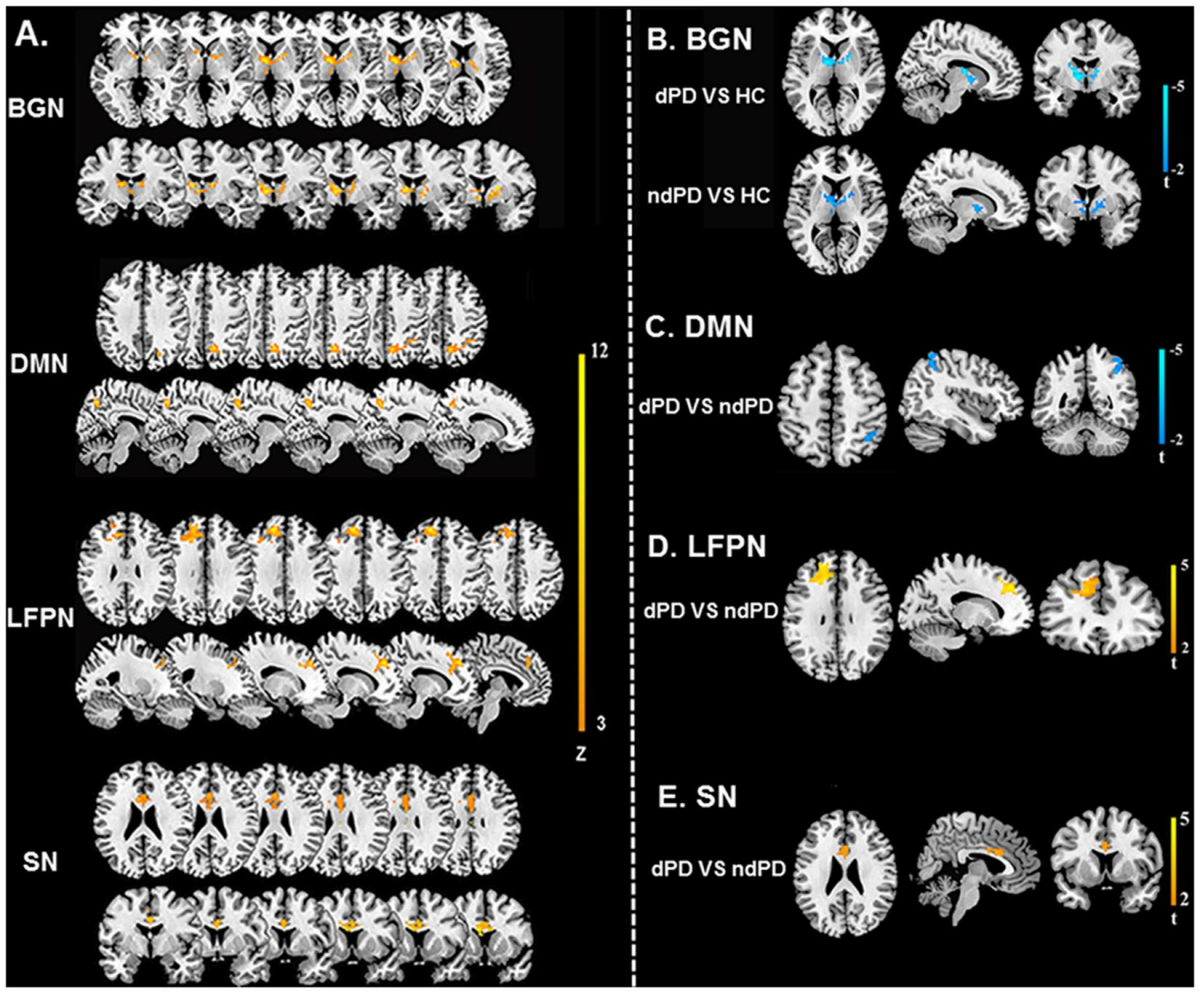
Inranetwork connectivity changes in dPD, ndPD and HC. (**A**) The ANOVA results for abnormal inranetwork connectivity among dPD, ndPD and HC groups (one-way ANOVA, p < 0.05, topoFDR corrected). (**B**), (**C**), (**D**) and (**E**) were the results for post-hoc comparison of inranetwork connectivity in dPD, ndPD and HC. (**B**) BGN showed decreased connectivity in dPD and ndPD by comparing with HC. (**C**) DMN displayed decreased connectivity in dPD compared with ndPD. (**D**) LFPN exhibited increased connectivity in dPD relative to ndPD. (**E**) SN showed increased connectivity in dPD compared to ndPD. Brian regions with cool (warm) color indicated significant decreased (increased) connectivity (two-sample post hoc t-tests, p < 0.05, topoFDR corrected).

BGN, DMN, LFPN, and SN were found to display altered functional connectivity among the dPD, HC and ndPD groups (ANOVA, p < 0.05, topological false discovery rate (topoFDR)^27^ corrected, Fig. 2, Table 2). Two-sample post hoc t-tests were then applied to determine connectivity changes between each pair of the three groups (p < 0.05, topoFDR corrected, Fig. 2, Table 3). Compared with HC, dPD and ndPD patients showed decreased connectivity in the BGN (e.g. putamen, caudate, and thalamus). Comparison of dPD and ndPD subgroups, dPD patients had increased connectivity in the LFPN (e.g. DLPFC) and SN (e.g. ACC), and decreased connectivity in the DMN (e.g. LPC).

**Table 2:** Comparisons of intranetwork connectivity among dPD, ndPD and HC groups (one-way ANOVA, p < 0.05, topoFDR corrected).

Anatomic region	Side	BA	Cluster Size	MNI coordinates			Z value
				x	y	z	
**BGN**							
Thalamus	R	—	17	9,	−3,	6	5.01
Thalamus	L	—	45	−12,	6,	12	6.32
Caudate	R	—	10	12,	−6,	15	5.46
Caudate	L	—	9	−12,	12,	0	4.25
Putamen	R	—	20	21,	6,	9	5.81
Putamen	L	—	13	−24,	9,	3	4.95
**DMN**							
PreCU	R	7	82	9,	−63,	48	11.65
LPC	R	40	56	42,	−48,	54	6.68
**FPN**							
DLPFC	L	9	170	−12,	39,	39	9.52
**SN**							
ACC	R	24	143	3,	21,	27	9.81

L, left; R, right; BA, Brodman area; MNI, Montreal Neuroscience Institute template; BGN, basal ganglia network; DMN, default-mode network; FPN, frontoparietal network; SN, salience network; LPC, Lateral parietal cortex; DLPFC, dorsolateral prefrontal cortex; ACC, anterior cingulate cortex.

**Table 3:** Post-hoc comparison of intranetwork connectivity between dPD, ndPD and HC groups (two-sample post hoc t-tests, p < 0.05, topoFDR corrected).

Anatomic region	Side	BA	Cluster Size	MNI coordinates			T value
				x	y	z	
dPD vs HC							
**BGN**							
Thalamus	R	—	13	12,	−6,	12	−3.28
Thalamus	L	—	31	−12,	−6,	12	−3.54
Caudate	R	—	9	9,	9,	3	−2.03
Caudate	L	—	7	−12,	12,	0	−2.89
Putamen	R	—	21	21,	3,	9	−3.24
Putamen	L	—	13	−12,	9,	−3	−2.73
ndPD vs HC							
**BGN**							
Thalamus	R	—	45	18,	−15,	15	−2.92
Thalamus	L	—	27	−6,	15,	9	−3.13
Caudate	R	—	9	9,	9,	3	−2.28
Putamen	R	—	19	21,	6,	9	−2.66
Putamen	L	—	17	−24,	9,	3	−3.11
dPD vs ndPD							
**FPN**							
DLPFC	L	9	139	−12,	42,	39	4.36
**DMN**							
LPC	L	40	48	36,	−51,	51	−3.38
**SN**							
ACC	R	24	108	3,	15,	27	3.00

L, left; R, right; BA, Brodman area; MNI, Montreal Neuroscience Institute template; BGN, basal ganglia network; DMN, default-mode network; FPN, frontoparietal network; SN, salience network; LPC, lateral parietal cortex; DLPFC, dorsolateral prefrontal cortex; ACC, anterior cingulate cortex.

At the 70-component level, 7 components were identified as the most representative ICNs for BGN, DMN, LFPN, RFPN, and SN. The DMN was represented in 3 components^28, 29^, including anterior DMN (aDMN; MPFC), inferior-posterior (ipDMN; PCC), and superior-posterior DMN (spDMN; bilateral precuneus and angular gyrus). The BGN, SN, LFPN, and RFPN were represented in one component, respectively. The selected ICNs were shown as Supplementary Fig. S1. With respect to 28-component level, the similar changes were discovered within these networks among dPD, ndPD, and HC groups for 70-component level (p < 0.05, uncorrected, see Supplementary Table S1, Fig. S2). Nonetheless, these results did not survive after cluster-level FDR (topoFDR) correction. This may attribute to the fact that component’s spatial features, volume, and mean z-score will change significantly as a function of model order^20^. The different spatial features and z-score distribution for the chosen components can affect the subsequent cluster-level FDR correction.

### Internetwork connectivity analysis

**Figure 3:**
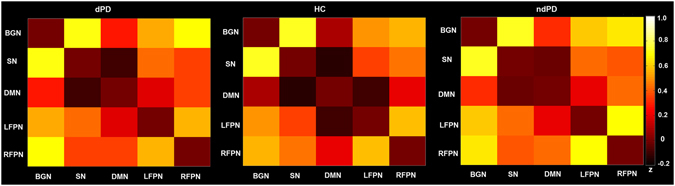
Internetwork connectivity matrix for dPD, HC and ndPD. Pairwise Pearson’s correlations between time courses of selected ICNs (BGN, DMN, LFPN, RFPN, and SN) were Fisher-z-transformed, averaged across subjects for each group, and presented in a correlation matrix. Colors represent intensity of averaged z-scores.

At the 28-component level, connectivity between BGN and DMN, and between bilateral FPN and DMN were altered among the three groups (Fig. 3, Table 4). The post-hoc analysis showed that (1) dPD patients compared to HC had increased connectivity between DMN and LFPN; (2) ndPD patients in contrast to HC had increased connectivity between DMN and bilateral FPN, and between BGN and DMN; (3) no significant differences were found among dPD and ndPD patients (Table 4). At the 70-component level, we found that (1) dPD patients compared with HC exhibited increased connectivity between aDMN and LFPN, as well as decreased connectivity between ipDMN and RFPN; (2) ndPD patients relative to HC exhibited increased connectivity between aDMN and bilateral FPN, and between BGN and aDMN; (3) dPD and ndPD patients did not show any significant differences. The similar results were obtained using the two ICA decomposition methods. More details about the results of ICA dimensionality = 70 were presented in Supplementary Table S2 (see Supplementary Fig. S3).

**Table 4:** Internetwork connectivity in dPD, ndPD, and HC groups.

Internetwork FC	dPD	HC	ndPD	p value
Anova				
BGN-SN	0.70 ± 0.42	0.72 ± 0.39	0.73 ± 0.31	0.96
BGN-DMN	0.27 ± 0.36	0.096 ± 0.27	0.32 ± 0.34	*0.015*
BGN-LFPN	0.55 ± 0.29	0.50 ± 0.42	0.60 ± 00.37	0.55
BGN-RFPN	0.69 ± 0.34	0.55 ± 0.27	0.65 ± 0.32	0.21
SN-DMN	−0.09 ± 0.48	−0.14 ± 0.34	−0.03 ± 0.38	0.48
SN-LFPN	0.42 ± 0.43	0.36 ± 0.46	0.43 ± 0.36	0.75
SN-RFPN	0.35 ± 0.33	0.44 ± 0.36	0.38 ± 0.39	0.66
DMN-LFPN	0.19 ± 0.43	−0.09 ± 0.31	0.21 ± 0.44	*0.004*
DMN-RFPN	−0.35 ± 0.33	−0.20 ± 0.30	−0.43 ± 0.46	*0.047*
RFPN-LFPN	0.56 ± 0.33	0.59 ± 0.40	0.70 ± 0.35	0.30
Post-hoc comparison				
BGN-DMN	dPD vs HC (p = 0.17)	ndPD vs HC (*p* = 0.015*)	dPD vs ndPD (p = 1.0)	
DMN-LFPN	dPD vs HC (*p* = 0.04*)	ndPD vs HC (*p* = 0.006*)	dPD vs ndPD (p = 1.0)	
DMN-RFPN	dPD vs HC (p = 0.52)	ndPD vs HC (*p* = 0.043*)	dPD vs ndPD (p = 1.0)	

The values for each group are denoted by the mean and standard deviation of connectivity value. Italics indicate p < 0.05; *Significant for p < 0.05, Bonferroni corrected for multiple comparisons. Abbreviations: BGN, basal ganglia network; DMN, default-mode network; LFPN, left frontoparietal network; RFPN, right frontoparietal network; SN, salience network.

At the 70-component level, we also used the identified 26 components (see Supplementary Fig. S4) to further verify the internetwork connectivity. The results were acquired by using the network-based statistic (NBS) method^30^ and post-hoc analysis. No significant differences were found between dPD and ndPD patients, and dPD patients relative to healthy subjects had altered connectivity between DMN and LFPN, in line with the above mentioned results obtained using the interested 5 or 7 components. Besides, compared to healthy controls, both dPD and ndPD patients had abnormal connectivity between BGN, sensorimotor, auditory, visual, and frontal networks. These results may explain the motor, visual, and auditory disturbance exhibited by all PD patients. More details were presented in Supplementary Table S3 (see Supplementary Figs S5 and S6).

### Correlation analysis

**Figure 4:**
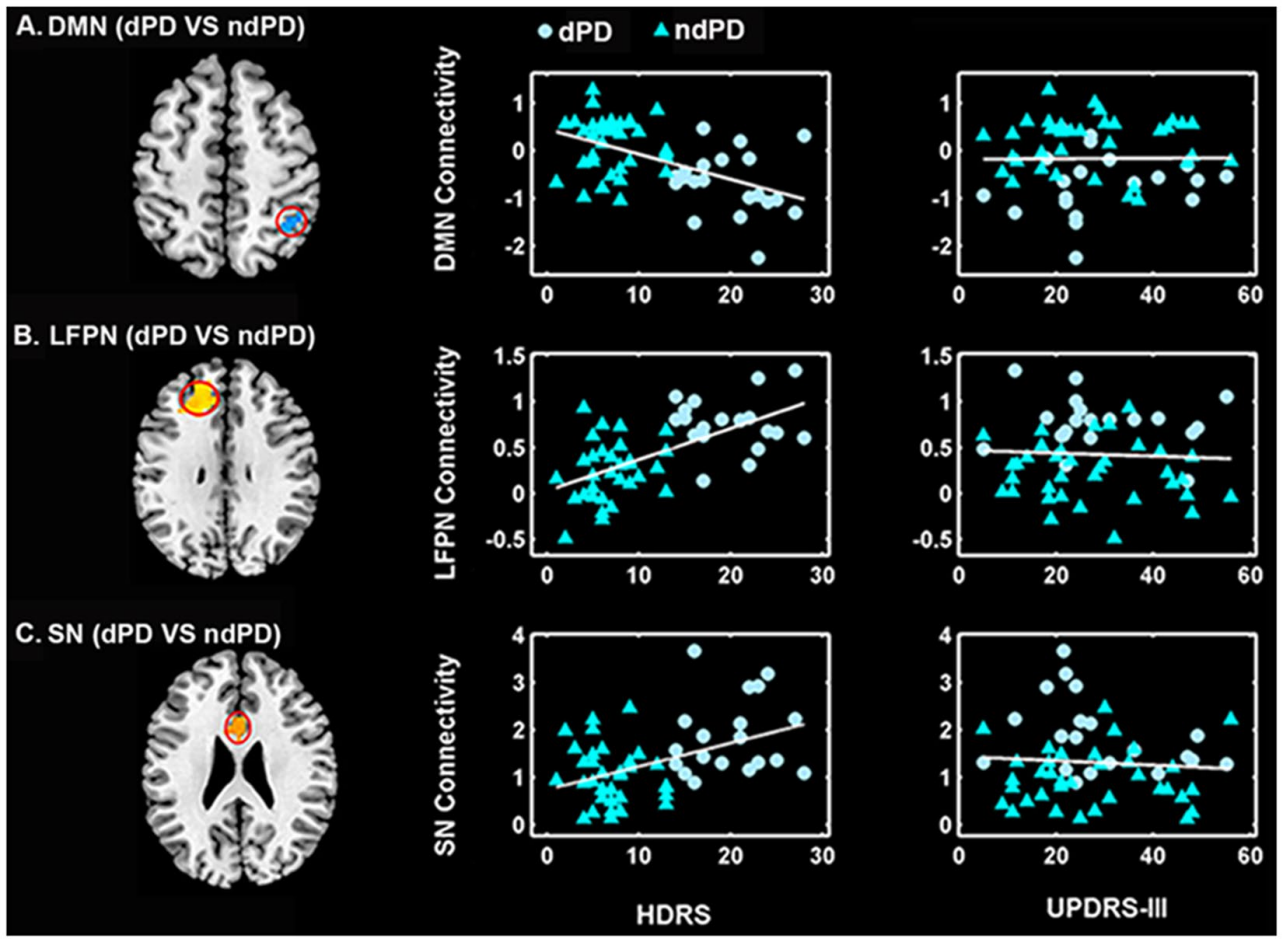
The correlations between intranetwork connectivity abnormities and the severity of depression and motor symptoms in PD. (**A**) DMN connectivity correlated negatively with HDRS scores (p < 0.001, r = −0.48), and uncorrelated with UPDRS-III (p = 0.98, r = −0.004) scores in PD. (**B**) LFPN connectivity correlated positively with HDRS scores (p < 0.001, r = 0.58), and uncorrelated with UPDRS-III (p = 0.96, r = 0.008) scores in PD. (**C**) SN connectivity correlated positively with HDRS scores (p = 0.004, r = 0.38), and uncorrelated with UPDRS-III (p = 0.89, r = −0.02) scores in PD.

Significant intranetwork connectivity differences among three groups were detected on the 28-component level (survived after topoFDR correction), and then these results were applied to correlated with clinical severity in patients with PD. Connectivity in the detected regions of BGN, DMN, LFPN, and SN were used for correlation analysis with the UPDRS-III and HDRS scores in all PD patients. The results showed that (1) connectivity in the BGN was uncorrelated with the UPDRS-III and HDRS scores; (2) connectivity in the DMN was correlated negatively with the HDRS scores, and connectivity in the LFPN and SN was correlated positively with the HDRS scores; (3) connectivity in the DMN, LFPN and SN was uncorrelated with the UPDRS-III scores (Fig. 4). For patients with dPD, connectivity in the confirmed significant regions did not show any correlation with the UPDRS-III and HDRS scores. This may be owing to the small sample size in dPD group, and further studies would be needed to test the relationship between connectivity anomalies and the severity of depression in dPD.

Since dPD and ndPD patients did not show any significant differences in internetwork connectivity, the correlation analysis between internetwork connectivity and the UPDRS-III and HDRS scores were not performed on the whole PD group. Besides, we exam the correlation between DMN and LFPN connectivity and the UPDRS-III and HDRS scores in dPD subgroup, as dPD compared to HC exhibited enhanced connectivity between DMN and LFPN. Connectivity between DMN and LFPN was uncorrelated with the UPDRS-III (p = 0.15, r = −0.33) and HDRS scores (p = 0.41, r = 0.20) in dPD. Moreover, connectivity between aDMN and LFPN, and between ipDMN and RFPN, obtained using high model order ICA, were uncorrelated with the UPDRS-III (p = 0.73, r = 0.08; p = 0.75, r = −0.08) and HDRS scores (p = 0.45, r = −0.18; p = 0.98, r = 0.005) in dPD.

## Discussion

In the current study, we applied rs-fMRI combined with two ICA decomposition methods to explore intrinsic connectivity changes within and between large-scale neural networks in PD with depression. Similar results can be obtained using these two decomposition algorithms: 1) dPD and ndPD patients relative to healthy subjects had decreased BGN connectivity; 2) dPD patients compared with ndPD patients exhibited increased LFPN and SN connectivity, and decreased DMN connectivity; 3) dPD patients in contrast to healthy controls showed hyperconnectivity between DMN and LFPN. Furthermore, connectivity abnormities in the DMN, LFPN and SN correlated with the severity of depression in PD. Our results confirmed the BGN, LFPN, DMN, and SN dysfunction associated with depression in PD.

The BG subserves a wide range of functions, including motor, cognitive, motivational, and emotional processes, and disruption of this circuit has been implicated in numerous neurological and psychiatric disorders^31^. In patients with PD, the degeneration of dopaminergic neurons in the substantia nigra pars compacta triggers a cascade of functional changes affecting the whole BG network^32^. Dopaminergic changes in BG network are responsible for the development of the cardinal motor features in PD, such as tremor, rigidity, and akinesia^33^. However, the dopaminergic degeneration in BG circuit was not thought to be associated purely with motor control in PD. Previous studies with PET or SPECT have found that depressed PD patients are related to dopamine loss in the striatum^5, 9, 34^, indicating the role of BG dopaminergic circuit in the occurrence of depression in PD. Indeed, the BG is intimately connected with the cortex through several segregated but parallel loops, which have been subdivided into motor, associative (cognitive), and limbic (emotional) domains^35^. They deal with the control of movement, behavior and cognition, and reward and emotions^35^, respectively. Dysfunction of nonmotor BG circuit has been proposed to explain the mood disturbances exhibited by PD patients^36^. In the present study, we found dPD and ndPD patients had reduced BGN connectivity, demonstrating that functional disruption in BGN was a common pathological change in depressed and non-depressed PD patients, and reinforcing the view that the BG dopaminergic circuit plays an essential part in the pathogenesis of depression in PD.

The DMN is a constellation of brain regions characterized by functions of self-referential processes^37^, and impairment in this network contributes to the characteristic symptom of self-focused rumination in primary depression^38, 39^. Although the involvement of DMN in primary depression is well documented, its role in PD with depression remains uncertain. To our knowledge, only three rs-fMRI studies have delineated the role of DMN in depression in PD. Two rs-fMRI studies reported that dPD exhibited abnormalities in the nodes of DMN, such as decreased ALFF value in the ventral MPFC^14^, and increased eigenvector centrality (EC) value in the PCC^40^, supporting the DMN impairment related to depression in PD. While another rs-fMRI study found that both dPD and ndPD patients had connectivity alterations in the DMN (e.g. PCu)^12^, demonstrating the DMN dysfunction is a common pathological condition in PD. A former literature provided further evidence for the above viewpoint by showing that there was an early functional disruption of the DMN in cognitively unimpaired PD patients^19^. Taken together, previous findings on the DMN dysfunction in depression in PD are contradictory. Here we found dPD patients had reduced DMN connectivity (e.g. LPC) relative to ndPD patients, in accordance with previous two rs-fMRI studies^14, 40^, lending support to the DMN dysfunction relevant to depression in PD. The current result may indicate that depressed PD patients, like patients with primary depression, had a failure to normally regulate self-referential activity due to the DMN’s impairment^41^. As mentioned above, conflict findings are reported with respect to the role of DMN in PD with depression, and thus the present result of abnormal DMN connectivity involved in depressed PD should be further validated.

The FPN consisting of DLPFC and PPC takes the charge of top-down regulation of attention and emotion^42^. Abnormal communication within the FPN may underlie deficits in cognitive control and emotional regulation in primary depression^42^. The DLPFC including portions of the middle and superior frontal gyrus on the lateral surface of the frontal lobes is a key component in FPN, and dysfunction of this area has been recognized as a hallmark for the pathophysiology of depression in PD^8, 14, 40, 43–46^. SPECT and PET studies found that dPD patients exhibited increased blood flow^45^ and serotonin transporter density^44^ in the DLPFC. Two rs-fMRI studies reported that dPD patients had reduced ALFF^14^ and EC^40^ value in the DLPFC. Moreover, the DLPFC was identified as a potential therapeutic target for dPD patients^43^. Those findings demonstrate the association of DLPFC dysfunction with depression in PD. This study found dPD patients exhibited increased connectivity in the DLPFC, adding to the growing evidence for the DLPFC disruption devoted to the presence of depression in PD. According to former literature, dysfunction of the DLPFC involved in dPD patients may be secondary to pathology in the dopaminergic projections from the ventral tegmental area (VTA) to the prefrontal cortex^46, 47^. Consequently, dopamine depletion in the mesocortical pathway could be used to explain the altered DLPFC connectivity with dPD in our study. Since the DLPFC anomalies are secondary to prefrontal dopaminergic deficiency, patient’s dopaminergic state (“ON” or “OFF”) would influence connectivity patterns in this region. Two fMRI studies have demonstrated administration of levodopa relatively normalized the DLPFC connectivity in PD^48, 49^. This is one possible cause for the DLPFC changes in opposite direction in our study (“ON” state) compared with previous two rs-fMRI studies (“OFF” state)^14, 40^. Another possible cause is that ICA-derived functional connectivity is distinct from the ALFF and EC index. ALFF approach measures regional cerebral activity changes^12^. EC technique is similar to node degree, aimed to identify a prominent or diminished role of a specific region in whole brain network^50^, which is differ from the ICA method aimed to divide the whole brain into several subnetworks.

The SN with its robust connections to several limbic and subcortical structures has been conceptualized as a bottom-up processor of salient experiences^26^. Abnormalities of SN lead to an impaired salience processing, contributing to the onset and maintenance of depressive symptom in general population^51, 52^. Investigators have found greater activation in depressed than in non-depressed subjects in the nodes of SN (e.g. insula and ACC) across a wide range of negative conditions^53–55^. The SN involved in PD with depression has also been demonstrated in several studies. For instance, previous PET studies revealed ACC hypometabolism associated with depression in PD^46, 56^. Depressed PD patients exhibited a specific loss of dopamine and noradrenaline innervation in the ACC^6, 57^. In addition, microstructural changes in the ACC bundles were related to dPD patients^58, 59^. In this study, depressed PD patients had increased connectivity in the ACC, adding to evidence that ACC could play a major role in the pathogenesis of depression in PD. The involvement of ACC dysfunction in depressed PD, as described in former research, can attribute to the degeneration of the mesolimbic dopaminergic system in dPD patients^6, 57^. The current result of increased ACC connectivity in dPD patients, thus, may ascribe to their mesolimbic dopaminergic dysfunction. Within the SN, an anomalous function of the ACC is among the most consistent findings in depressed PD patients. The question of whether other primary nodes of the SN (e.g. anterior insula) affected in dPD needs to be further investigated.

In addition to the above mentioned intranetwork connectivity abnormities associated with dPD, dPD patients had enhanced connectivity between LFPN and DMN compared to healthy subjects. Specially, we found that the correlation between DMN and LFPN was negative in healthy controls but positive in dPD patients, indicating that normal reciprocity (anticorrlation) between DMN and FPN was weakened in dPD patients. This weakened reciprocity would lead to a poor switching between DMN-based self-referential and FPN-based goal-directed processes in primary depression, accounting for depressive symptoms of patients with this disease^42, 60^. Here we discovered reduced anticorrelation between DMN and LFPN in dPD, in keeping with previous findings on patients with primary depression, suggesting inappropriate engagement of anticorrelated networks is a generic marker for the development of depressive symptoms. Abnormal communication between DMN and FPN implicated in dPD can be also validated by using high model order ICA, even though the relationship between DMN’s subsystem and FPN was partially reorganized. These reorganized internetwork connectivity patterns found in our sample correspond with previous high model order ICA studies^28, 29^. The current findings needs to be further testified due to the lack of study on the relationship between ICNs in dPD.

Finally, we found that connectivity anomalies in the BGN did not correlate with the HDRS and UPDRS-III scores (depressive and motor severity) in PD patients, in line with a previous rs-fMRI study presenting findings on BGN connectivity having no relationship with clinical indices of severity in PD^17^. This may indicate that BGN connectivity, similar to substantia nigra hyperechogenicity as identified by transcranial sonography^61^, is a trait and not a state biomarker of disease. Connectivity abnormalities in the DMN, LFPN and SN correlated with the HDRS scores but not with the UPDRS-III scores in PD patients, suggesting that dysfunction of the DMN, SN, and LFPN is responsible for the presence of depressive symptoms rather than motor symptoms in PD, and reinforce the hypothesis of functional disruption of the DMN, LFPN and SN involved in depression in PD.

Several limitations warrant attention and suggest directions for future research. First, our selection of high model order ICA was empirical. Although it has been demonstrated that 70 components seems to be an optimal choice^20^, computational or objective criterion is still missing. To alleviate the impact of model order selection on the results, we also conducted analysis at 28-component level based on the minimum description length criteria^21^. Second, the dPD (n = 20) patients sample size is relatively small. Nevertheless, dPD patients were enrolled using stringent inclusion criteria, such as recruitment of mild to moderate stage patients, no use of antidepressants and dopamine agonists, etc. Third, although the MMSE was used to exclusion of demented patients, mild cognitive impairment in patient sample cannot be fully ruled out. This issue, however, may not influence our results a great deal, since dPD and ndPD patients did not differ in terms of their MMSE scores. The Montreal Cognitive Assessment scale (MoCA) that is preferred over the MMSE for screening of mild cognitive dysfunction could be included in future studies. Fourth, the use of medication is an important confounder. However, both groups of patients were taking similar doses of dopaminergic drugs (comparison of LED between dPD and ndPD, p = 0.71). Moreover, we performed correlation analysis between the LED and altered functional connectivity in the DMN, LFPN, and SN, and did not reveal any significant association (DMN: p = 0.27, r = 0.15; LFPN: p = 0.15, r = −0.20; SN: p = 0.11, r = 0.22). Finally, the lack of non-PD depression group left unanswered the question whether dysfunctional neural networks (e.g. BGN, DMN, FPN, and SN) observed in depressed PD patients were associated with depression in the general population. It does not enable us to determine whether the depression of PD shares a common neurobiological substrate with that of primary depression. Individuals with primary depression would be recruited in the future to help us better understanding of the dysfunctional neural networks in depression, PD with depression, and PD without depression.

In summary, this study, to best of our knowledge, provides the first evidence of neural network dysfunction in depressed PD patients, including reduced connectivity in the BGN and DMN, increased connectivity in the LFPN and SN, as well as hyperconnectivity between DMN and LFPN. These neuroimaging deficits exhibited by dPD patients may attribute to dysfunction of BG dopaminergic circuits and mesocorticolimbic dopamine systems in dPD^3, 57, 62^. However, this hypothesis should be evaluated by future rs-fMRI studies combined with the corresponding PET/SPECT data.

## Methods

### Participants

70 right-handed PD patients (21 dPD and 49 ndPD) were recruited from the movement disorders outpatient clinic of Nanjing Brain Hospital (Nanjing, China). All had a diagnosis of idiopathic PD by an experienced neurologist according to the UK Parkinson Disease Society Brain Bank Criteria. To minimize the impact of head motion, PD patients were studied while taking their usual medications (“ON” state). Exclusion criteria were: (1) moderate to severe head tremor; (2) cerebrovascular disorders, including previous stroke, history of head injury, history of seizure, hydrocephalus, intracranial mass, previous neurological surgery and other neurologic diseases; (3) antiparkinsonian treatment with dopamine agonists, (4) antidepressant treatment or other psychiatric therapy; (5) Mini Mental State Examination (MMSE) score < 24; and (6) incomplete clinic information. 4 ndPD patients were excluded from the present analyses due to the stringent exclusion criteria. The remaining 66 PD patients were on stable dopaminergic treatment for at least 4 weeks prior to study entry. In addition, 50 right-handed healthy controls (HC) were recruited from local individuals who volunteered to participate in scientific studies. 1 HC with MMSE <24 were discarded. The remaining 49 control subjects had a normal neurological status with no history of either neurological or psychiatric diseases. Table 1 contains additional demographic details. The study was carried out in accordance with the Declaration of Helsinki. Experimental protocols were approved by the medical research ethical committee of Nanjing Brain Hospital. Written informed consent was obtained from all participants.

### Neuropsychological Evaluation

Psychometric and neurologic assessments with all PD patients were done in the “ON” state, i.e. with their usual antiparkinsonian medication. Each patient’s disease severity was measured by H&Y stage and UPDRS-III. Only mild to moderate stage patients were enrolled in the study in order to complete a long scan. Non-depressed patients were matched with depressed patients on the basis of disease severity. Diagnosis of depression was using the Diagnostic and Statistical manual of Mental Disorders, Fifth Edition (DSM-V) criteria by an experienced, board-certified psychiatrist trained for Structured Clinical Interview. The severity of depression was evaluated using the 17-item Hamilton Depression Rating Scale (HDRS-17). All depressed PD patients had a HDRS-17 score higher than 14 points. All the subjects were administered the MMSE, and individuals with MMSE score <24 were not included. Data pertaining to age, gender, handedness, education level, disease duration, and clinical symptom ratings were collected by a movement disorder specialist prior to MRI examination.

### Image data acquisition

All the patients were in the “ON” state before and during scanning. Image data were acquired using a Siemens 3.0-Tesla signal scanner (Siemens, Verio, Germany). Subjects were instructed to stay awake and close their eyes, and to try not to think of anything. Functional imaging data were collected transversely by using a gradient-recalled echo-planar imaging (GRE-EPI) pulse sequence with the following settings: TR/TE = 200 ms/30 ms, flip angle = 90°, matrix = 64 × 64, FOV = 220 mm × 220 mm, thickness/gap = 3.5 mm/0.6 mm, in-plane resolution = 3.4 mm × 3.4 mm, slices = 31. For each subject, a total of 140 volumes were obtained, resulting in a total scan time of 280 s. High resolution anatomical images were acquired using a T1 fluid attenuated inversion recovery (FLAIR) sequence (TR/TE = 2530 ms/3.34 ms, flip angle = 7°, matrix = 256 × 192, FOV = 256 mm × 256 mm, slice thickness/gap = 1.33 mm/0.5 mm, 128 slices covered the whole brain).

### Data preprocessing

Structural images were reoriented to the anterior commissure and segmented into grey matter (GM), white matter (WM), cerebrospinal fluid (CSF), skull, and soft tissue outside the brain, using the standard segmentation option in SPM 12 (http://www.fil.ion.ucl.ac.uk/spm/). Then the segmented tissue class images (e.g., GM, WM) were employed to generate a group-specific template (across all subjects) using DARTEL toolbox in SPM12. The subject-specific flow fields yielded from the DARTEL procedure can be applied to corresponding functional data in the next stage.

Resting-state functional images preprocessing was carried out using both SPM12 and AFNI (http://afni.nimh.nih.gov/afni) packages. The steps including slice acquisition correction, head motion correction, spatial normalization, and smoothing were performed with SPM 12, and the dispike procedure was achieved in AFNI software. Briefly, the first 5 volumes were discarded to allow for magnetization stabilization. The remaining 135 consecutive images were then corrected for the acquisition delay between slices using the middle slice as the reference frame and further realigned to the first volume to correct for head movement with SPM12. 6 subjects (3 HC and 3 ndPD) with head motion exceeding ± 2.5 mm of translation or ± 2.5 degrees of rotation were excluded from the dataset. To minimize the impact of motion artifact on functional connectivity analysis^63^, 20 subjects (1 dPD, 7 ndPD, and 12 HC) with excessive instantaneous head motion (mean framewise displacement (FD) exceeding 0.3) were discarded, resulting 20 dPD, 35 ndPD, and 34 HC for the following analyses. The instantaneous head motion was calculated using the six head realignment parameters, as described in^63^. The motion-corrected volumes were then despiked using AFNI’s 3dDespike algorithm to mitigate the impact of outliers. The mean functional image across all realigned volumes was coregistered with the structural image, and the resulting warps applied to all the despiked functional volumes by utilizing SPM12. Finally, all the coregistered functional images were nonlinearly normalized, subject by subject, to the sample-specific group template (using subject-specific flow fields), affine-aligned into stereotactic space of the Montreal Neurological Institute (3 mm isometric voxel size), and smoothed using a 6 mm full-width at half maximum Gaussian filter^64^. In addition, six head motion parameters obtained in the realigning step, WM signal, CSF signal, and Legendre polynomials orders up to 2nd were included in a linear regression to remove possible spurious variances from the data. CSF and WM mean signals were determined by averaging the native-space functional time series of all voxels contained inside the corresponding masks obtained from the segmentation of the structural images using DARTEL.

### Intranetwork connectivity analysis

Preprocessed images were analyzed with the Group ICA of fMRI Toolbox (GIFT) software^65^, and following three main steps: (1) data reduction, (2) group ICA, and (3) back reconstruction. First, principal components analysis (PCA) was applied to reduce the data dimensionality for each subject. The reduced data from all subjects were then concatenated and entered into a second data reduction step using PCA. Second, the reduced, group concatenated data were entered into the ICA algorithm (Infomax)^66^ to calculate spatially independent group components. The number of independent group components was set at 28 and 70 respectively, based on the minimum description length criterion described in^23^ and high model order ICA used in^22^. The reliability of the independent components decomposition was tested by running Infomax 20 times in the ICASSO toolbox^67^. Third, individual subject components were back reconstructed from the group components using GICA approach^23, 65^, during which the aggregate components and the results from data reduction step were used to compute the individual subject components. Each back-reconstructed component consists of a spatial z-map reflecting component’s functional connectivity pattern across space and an associated time course reflecting component’s activity across time. The group-level components corresponding to BGN, DMN, SN, and bilateral FPN, were selected by visual inspection and confirmed using the template-matching procedure^68^. The template for BGN, DMN, SN, and FPN was provided in GIFT software (the RSN template), and the map of each component was spatially correlated with specific network template. The component with largest spatial correlation coefficients with each of these templates was chosen and reconfirmed by visual inspection. To further confirm our selected ICNs, we also generated the network templates using the WFU Pickatlas^69^ on the basis of the Brodmann areas and cluster peaks reported in the literature (DMN^70^; SN^26^; FPN^71^; BGN^17^). Spatial correlation was performed between the components and the generated templates. For high model order ICA, the RSN template, the generated network template, and the template came from online T-maps of 28 components^29^ (http://mialab.mrn.org/data/hcp/RSN_HC_unthresholded_tmap s.nii) was employed to match with our 70 independent components’ spatial maps. The subsystems of BGN, DMN, FPN, and SN were chosen based on the largest spatial correlation with these templates. Functional connectivity within each selected ICN was calculated using the reconstructed component’s spatial z-maps.

### Internetwork connectivity analysis

To evaluate functional connectivity between the selected ICNs, subject specific ICN’s time courses were detrended, despiked, filtered using a fifth-order Butterworth low-pass filter with a high frequency cutoff of 0.15 Hz, and pairwise correlated by Pearson’s correlation, following the approach of Jafri and colleagues^23^. Correlation coefficients were then transformed to z-scores using Fisher’s z-transformation. At the 28-component level, the number of pair-wise combinations is 10 for each subject as 5 components were identified. At 70-component level, 7 components were identified as the most representative ICNs for BGN, DMN, LFPN, RFPN, and SN, and the number of pair-wise combinations is 21 for each subject.

To further verify the internetwork connectivity, we also used the identified 26 components derived from 70 components to conduct the internetwork connectivity analysis. The identified 26 components reflected the BGN, DMN, auditory, visual, sensorimotor, attention, and frontal networks, respectively^29^. The NBS method and post-hoc analysis was applied to determine connectivity changes between three groups.

### Statistical analysis

Differences between groups in terms of demographic and clinical variables were conducted by Pearson chi-square test, One-way analysis of variance (ANOVA), Wilcoxon rank sum test, and Student t test in SPSS software package (SPSS Inc, Chicago, Illinois, USA), as appropriate. The level of statistical significance was set at P < 0.05.

To statistically evaluate functional connectivity within each selected ICN, we calculated voxel-wise one-sample t-tests on participants’ reconstructed spatial maps for each group using SPM12 (p < 0.001, false discovery rate (FDR) corrected). Comparisons of connectivity within each ICN among dPD, ndPD, and HC groups were performed by using a design model of one-way ANOVA in SPM12, followed by post-hoc two-sample t tests. The significance threshold was set at p < 0.05, corrected for multiple comparisons using topoFDR in SPM12. Between-group differences of connectivity among selected ICNs were assessed using an ANOVA model in SPSS, and post-hoc two-sample t tests were carried out to determine connectivity changes between each pair of the three groups (p < 0.05, Bonferroni corrected).

The correlations between the detected connectivity abnormalities and the HDRS, UPDRS-III scores were assessed for overall PD and dPD patients respectively, by using Spearman correlation coefficient. The statistical level with P < 0.05 was considered as significant.

## Electronic supplementary material

Aberrant Intra- and Internetwork Functional Connectivity in Depressed Parkinson’s Disease
