# Supplementary material for: Aberrant Intra- and Internetwork Functional Connectivity in Depressed Parkinson’s Disease
Source: Sci Rep. 2017 May 31;7:2568. doi: 10.1038/s41598-017-02127-y (PMC5451438; doi:10.1038/s41598-017-02127-y)
Supplement: Supplementary file 1 — Aberrant Intra- and Internetwork Functional Connectivity in Depressed Parkinson’s Disease [file 41598_2017_2127_MOESM1_ESM.pdf]

Aberrant Intra- and Internetwork Functional Connectivity in Depressed Parkinson’s Disease

Luqing Wei, PhD, Xiao Hu, MD, Yajing Zhu, MD, Yonggui, Yuan, MD, Weiguo Liu, MD, Hong Chen, PhD

Supplementary Tables

Supplementary Table S1. Intranetwork connectivity changes in dPD, ndDP, and HC groups using ICA dimensionality of 70

Anatomic region	Side	BA	Cluster Size	MNI coordinates	T value
dPD vs HC					
**BGN**					
Putamen	R	-	21	30, 6, -3	-2.50
Putamen	L	-	63	-12, 0, 12	-2.92
**LFPN**					
PPC	L	7/40	85	-33, -48, 48	3.21
DLPFC	L	8/9	80	-39, 21, 48	3.34
**SN**					
ACC	R	32	56	9, 42, 3	2.93
ndPD vs HC					
**BGN**					
Thalamus	R	-	67	9, -9, 12	-2.97
Putamen	L	-	29	-18, 12, 6	-2.95
Putamen	R	-	27	33, 6, -3	-2.91
**ipDMN**					
PCC	L	30/29	122	-3, -63, 9	- 3.43
**LFPN**					
PPC	L	40/7	168	-30, -63, 51	3.49
**SN**					
ACC	R	32	94	12, 51, 9	2.81
dPD vs ndPD					
**spDMN**					
LPC	L	39	72	-39, -60, 27	-3.12
**LFPN**					
DLPFC	L	9/8	42	-18, 42, 36	3.76
**SN**					
ACC	R	32	75	6, 39, 15	3.00

Comparison of intranetwork connectivity between dPD, ndPD and HC groups (two-sample t-tests, $p<0.05$, uncorrected). L, left; R, right; BA, Brodman area; MNI, Montreal Neuroscience Institute template; BGN, basal ganglia network; LFPN, left frontoparietal network; RFPN, right frontoparietal network; SN, salience network; a/ip/spDMN, anterior/inferior-posterior/superior-posterior DMN; LPC, lateral parietal cortex; PPC, posterior parietal cortex; DLPFC, dorsolateral prefrontal cortex; PCC, posterior cingulate cortex; ACC,

anterior cingulate cortex.

Supplementary Table S2. Internetwork connectivity changes between BGN, aDMN, ipDMN, spDMN, LFPN, RFPN, and SN in dPD, ndPD, and HC groups using ICA dimensionality of 70

Internetwork FC	dPD	HC	ndPD	p value
**Anova**				
BGN-SN	0.37 ± 0.31	0.45 ± 0.25	0.48 ± 0.32	0.41
BGN-aDMN	0.30 ± 0.32	0.21 ± 0.35	0.42 ± 0.36	*0.048*
BGN-spDMN	0.34 ± 0.24	0.42 ± 0.26	0.35 ± 0.34	0.48
BGN-ipDMN	-0.19 ± 0.30	-0.33 ± 0.34	-0.18 ± 0.42	0.21
BGN-LFPN	0.28 ± 0.23	0.27 ± 0.30	0.34 ± 0.32	0.61
BGN-RFPN	0.49 ± 0.24	0.59 ± 0.24	0.32 ± 0.38	*0.002*
SN-aDMN	0.48 ± 0.39	0.48 ± 0.41	0.60 ± 0.39	0.42
SN-spDMN	0.61 ± 0.25	0.69 ± 0.35	0.62 ± 0.28	0.58
SN-ipDMN	-0.38 ± 0.25	-0.42 ± 0.40	-0.40 ± 0.41	0.92
SN-LFPN	0.29 ± 0.29	0.22 ± 0.32	0.30 ± 0.36	0.49
SN-RFPN	0.34 ± 0.27	0.22 ± 0.32	0.27 ± 0.42	0.48
aDMN-spDMN	0.81 ± 0.36	0.78 ± 0.36	0.80 ± 0.32	0.96
aDMN-ipDMN	-0.20 ± 0.48	-0.17 ± 0.47	-0.35 ± 0.52	0.27
aDMN-LFPN	0.61 ± 0 .43	0.32 ± 0.36	0.64 ± 0.40	*0.002*
aDMN-RFPN	0.04 ± 0.56	-0.20 ± 0.49	0.14 ± 0.47	*0.02*
ipDMN-spDMN	-0.21 ± 0.23	-0.40 ± 0.42	-0.34 ± 0.39	0.19
spDMN-LFPN	0.61 ± 0.32	0.43 ± 0.32	0.57 ± 0.34	0.08
spDMN-RFPN	0.07 ± 0.41	0.12 ± 0.32	0.006 ± 0.38	0.41
ipDMN-LFPN	-0.18 ± 0.34	-0.07 ± 0.34	-0.18 ± 0.45	0.41
ipDMN-RFPN	-0.60 ± 0.42	-0.35 ± 0.27	-0.40 ± 0.38	*0.047*
LFPN-RFPN	0.30 ± 0.50	0.35 ± 0.39	0.37 ± 0.39	0.85
**Post-hoc comparison**				
BGN-aDMN	dPD vs HC (p=1.0)	ndPD vs HC (*p=0.043**)	dPD vs ndPD (p=0.68)	
BGN-RFPN	dPD vs HC (p=0.74)	ndPD vs HC (*p=0.001**)	dPD vs ndPD (p=0.16)	
aDMN-LFPN	dPD vs HC (*p=0.03**)	ndPD vs HC (*p=0.003**)	dPD vs ndPD (p=1.0)	
aDMN-RFPN	dPD vs HC (p=0.32)	ndPD vs HC (*p=0.021**)	dPD vs ndPD (p=1.0)	
ipDMN-RFPN	dPD vs HC (*p=0.048**)	ndPD vs HC (p=1.0)	dPD vs ndPD (p=0.15)	

The values for each group are denoted by the mean and standard deviation of connectivity value. Italics indicate p<0.05; *significant for p<0.05, Bonferroni corrected for multiple comparisons. Abbreviations: BGN, basal ganglia network; LFPN, left frontoparietal network; RFPN, right frontoparietal network; SN, salience network; a/ip/spDMN, anterior/inferior-posterior/superior-posterior DMN.

Supplementary Table S3. Internetwork connectivity changes between the identified 26 components in dPD, ndPD, and HC groups using ICA dimensionality of 70

	Internetwork FC	dPD vs HC (p value)	ndPD vs HC (p value)	dPD vs ndPD (p value)
BGN-VIS	IC30 -- IC22	0.0002	0.0035	0.355
BGN-VIS	IC53 -- IC22	0.00007	0.0014	0.202
BGN-FRONT	IC47 -- IC22	0.0007	0.00006	0.999
AUD-VIS	IC27 -- IC17	0.021	0.0013	0.414
AUD-VIS	IC42 -- IC17	0.255	0.0012	0.050
AUD-VIS	IC53 -- IC17	0.064	0.00005	0.195
MOT-VIS	IC42 -- IC4	0.014	0.000006	0.164
MOT-VIS	IC9 -- IC18	0.173	0.0003	0.086
MOT-VIS	IC27 -- IC18	0.007	0.0009	0.782
MOT-VIS	IC42 -- IC18	0.023	0.0001	0.218
MOT-VIS	IC53 -- IC18	0.0007	0.00001	0.521
MOT-FRONT	IC47 -- IC18	0.005	0.00006	0.631
MOT-VIS	IC42 -- IC28	0.181	0.00015	0.058
MOT-VIS	IC53 -- IC39	0.006	0.0028	0.965
MOT-VIS	IC27 -- IC54	0.004	0.0005	0.818
MOT-VIS	IC42 -- IC54	0.037	0.0018	0.490
MOT-VIS	IC53 -- IC54	0.000007	0.000006	0.718
FRONT-MOT	IC47 -- IC54	0.0008	0.00007	0.940
FRONT-MOT	IC47 -- IC56	0.019	0.0009	0.594
VIS-VIS	IC42 -- IC31	0.208	0.00036	0.089
DMN-LFPN	IC63 – IC35	0.01	0.04	0.336

Supplementary Figures

Fig. S1. Spatial maps of BGN, aDMN, ipDMN, spDMN, LFPN, RFPN, and SN in dPD, HC, and ndPD group using ICA dimensionality of 70.

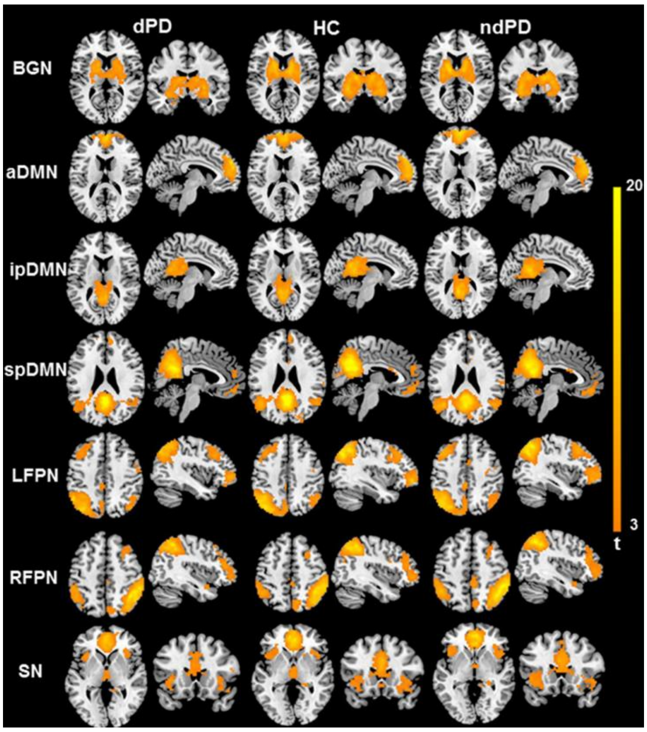

Spatial maps of BGN, aDMN, ipDMN, spDMN, LFPN, RFPN, and SN in dPD, HC, and ndPD group (one-sample t-test, $p<0.05$, FDR corrected). The first two columns represented brain maps for dPD, middle two columns represented brain maps for HC, and last two column represented brain maps for ndPD. Abbreviations: BGN, basal ganglia network; LFPN, left frontoparietal network; RFPN, right frontoparietal network; SN, salience network; a/ip/spDMN, anterior/inferior -posterior/superior-posterior DMN

7 components were selected as the most representative ICNs, which were matched previous results[1-4]. The BGN was represented in one component in accord with previous study on PD patients[1]. The SN was represented in one component, comprising ACC and bilateral anterior insular[2]. DMN was represented in 3 components[4,5] containing anterior DMN (aDMN; medial prefrontal cortex), inferior-posterior DMN (ipDMN; posterior cingulate cortex), and superior-posterior DMN (spDMN; bilateral precuneus and angular gyrus). LFPN and RFPN were represented in 2 components[3], comprising right/left posterior parietal cortex, and right/left DLPFC.

Fig. S2 Intranetwork connectivity changes in dPD, HC and ndPD using ICA dimensionality of 70

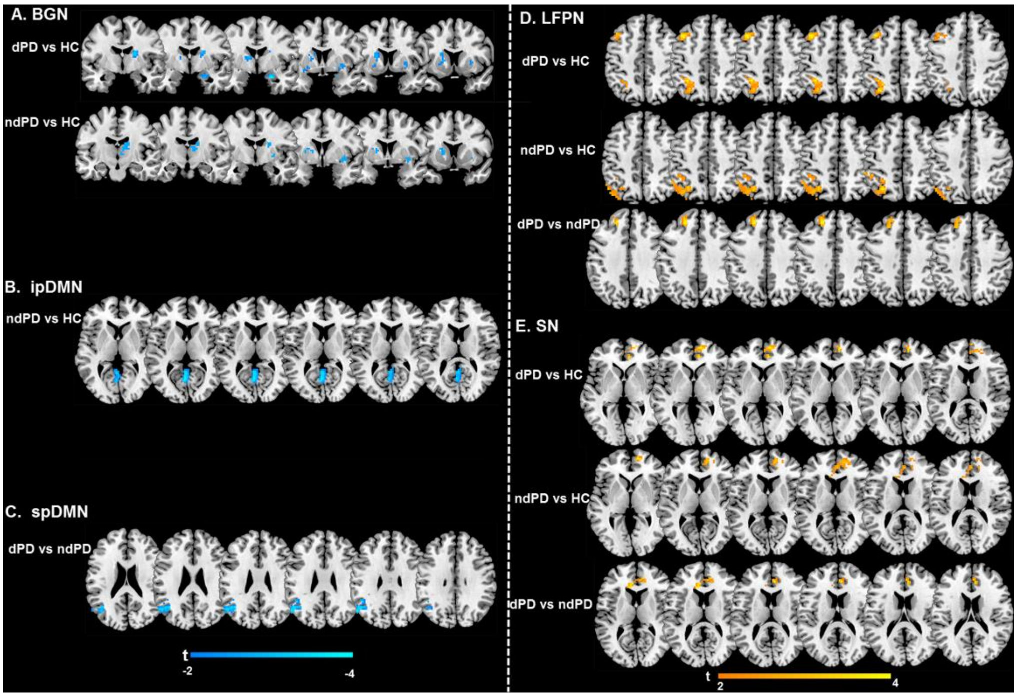

Intranetwork connectivity changes between dPD, ndPD and HC groups (two-sample t-tests, $p<0.05$, uncorrected). (A) BGN showed decreased connectivity in dPD and ndPD by comparing with HC. (B) ipDMN displayed decreased connectivity in ndPD compared with HC. (C) spDMN exhibited decreased connectivity in dPD relative to ndPD. (D) LFPN showed increased connectivity in dPD and ndPD by comparing with HC, and increased connectivity in dPD compared to ndPD. (E) SN displayed increased connectivity in dPD and ndPD by comparing with HC, and increased connectivity in dPD contrast to ndPD.

Fig. S3 Internetwork connectivity matrix for BGN, aDMN, ipDMN, spDMN, LFPN, RFPN, and SN in dPD, HC and ndPD using ICA dimensionality of 70

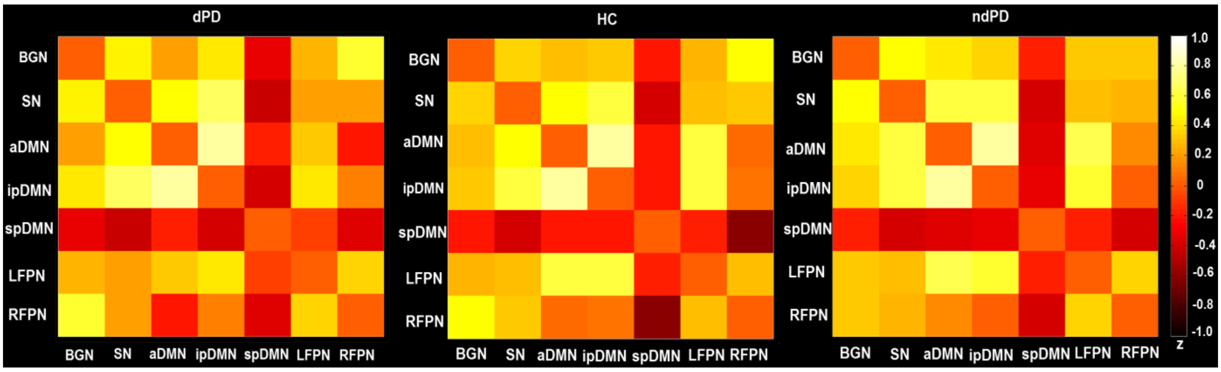

Pairwise Pearson’s correlations between time courses of selected ICNs (BGN, aDMN, ipDMN, spDMN, LFPN, RFPN, and SN) were Fisher-z-transformed, averaged across subjects for each group, and presented in a correlation matrix. Colors represent intensity of averaged z-scores. Abbreviations: BGN, basal ganglia network; LFPN, left frontoparietal network; RFPN, right frontoparietal network; SN, salience network; a/ip/spDMN, anterior/inferior -posterior/superior-posterior DMN.

Fig. S4. Spatial maps of the identified 26 components using ICA dimensionality of 70.

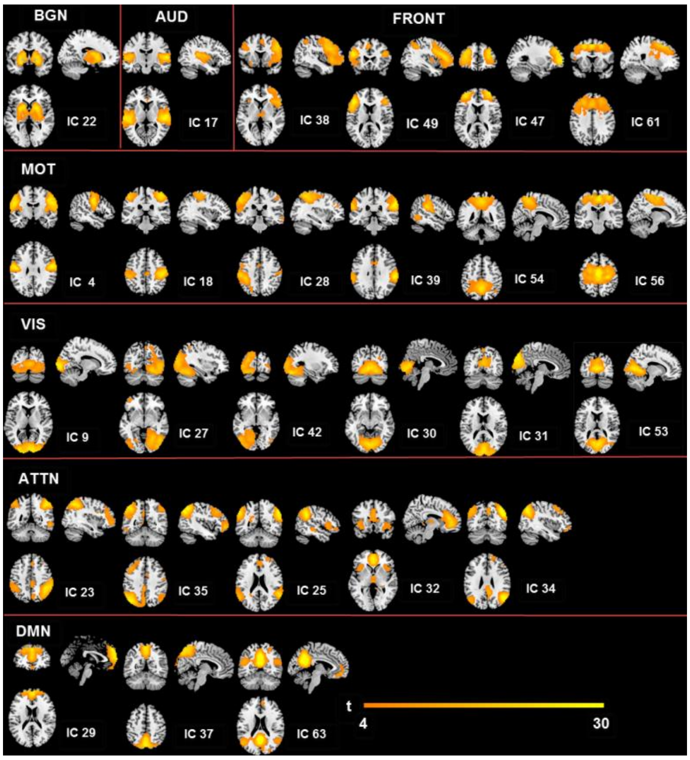

Spatial maps of BGN, DMN, AUD, FRONT, MOT, VIS, and ATTN network in all subjects (one-sample t-test, $p<0.001$, FDR corrected). Abbreviations: BGN, basal ganglia network; AUD, auditory; FRONT, frontal; MOT, sensorimotor; VIS, visual; ATTN, attention; DMN, default model network.

26 components were chosen as the most representative ICNs, in line with previous studies[4,5]. The BGN (IC 22) and AUD network (auditory, IC 17, bilateral superior temporal gyrus) was represented in one component, respectively. The FRONT (frontal) network was represented in 4 components, including IC 38 (right inferior/middle frontal gyrus), IC 49 (left inferior/middle frontal gyrus), IC 47 (bilateral superior/middle gyrus), and IC 61 (bilateral superior/middle gyrus, and bilateral cingulate gyrus). The MOT (sensorimotor) network was represented in 6 components, containing IC 4 (bilateral precentral gyrus), IC 18 (bilateral postcentral gyrus), IC 28 (left precentral/postcentral gyrus), IC 39 (bilateral supramarginal gyrus and middle cingulate cortex), IC 54 (bilateral paracentral lobe and postcentral gyrus), and IC 56 (supplementary motor area, bilateral paracentral lobe, and precentral/postcentral gyrus). The VIS (visual) network was represented in 6 components, comprising IC 9 (bilateral cuneus), IC 27 (right lingual gyrus, middle occipital gyrus, and cuneus), IC 42 (left lingual gyrus, middle occipital gyrus, and cuneus), IC 30 (bilateral lingual gyrus), IC 31 (bilateral cuneus, superior occipital gyrus, calcarine gyrus), and IC 53 (bilateral calcarine gyrus). The ATTN (attention) network was represented in 5 components, including IC 23 (RFPN, right posterior parietal cortex and right DLPFC), IC 35 (LFPN, left posterior parietal cortex and left DLPFC), IC 32 (SN, ACC and

bilateral anterior insular), IC 25 (bilateral inferior parietal lobe, bilateral middle temporal gyrus, and bilateral middle frontal gyrus), and IC 34 (bilateral angular gyrus/inferior parietal lobe, right superior/middle temporal gyrus, and right middle frontal gyrus). The DMN was represented in 3 components, containing IC 29 (aDMN, medial prefrontal cortex), IC 63 (ipDMN, posterior cingulate cortex), and (spDMN, bilateral precuneus and angular gyrus).

Fig. S5 Internetwork connectivity matrix for the identified 26 components in dPD, HC and ndPD using ICA dimensionality of 70

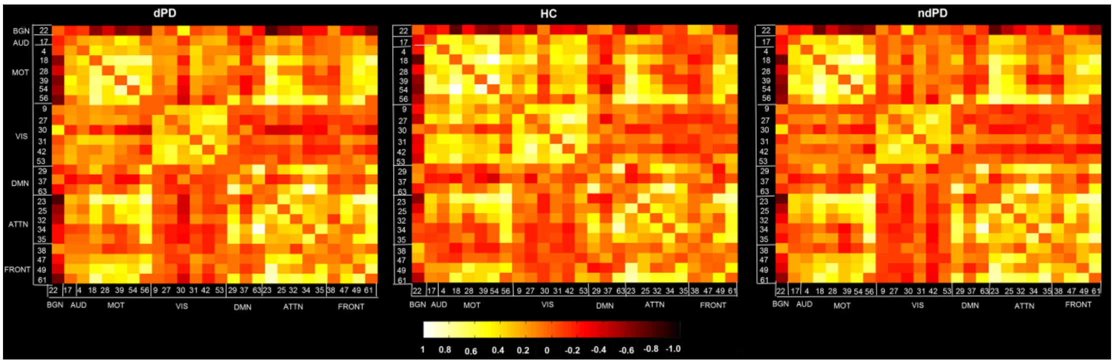

Pairwise Pearson's correlations between time courses of the selected 26 components were Fisher-z-transformed, averaged across subjects for each group, and presented in a correlation matrix. Colors represent intensity of averaged z-scores. The number in the x-axis and y-axis denoted the component's number (22, 17, 4, 18, 28, 39, 54, 56, 9, 27, 30, 31, 42, 53, 29, 37, 63, 23, 25, 32, 34, 35, 38, 47, 49, and 61). Abbreviations: BGN, basal ganglia network; AUD, auditory; FRONT, frontal; MOT, sensorimotor; VIS, visual; ATTN, attention; DMN, default model network.

Fig. S6 Internetwork connectivity changes between dPD, HC, and ndPD by using the network-based statistic (NBS) method

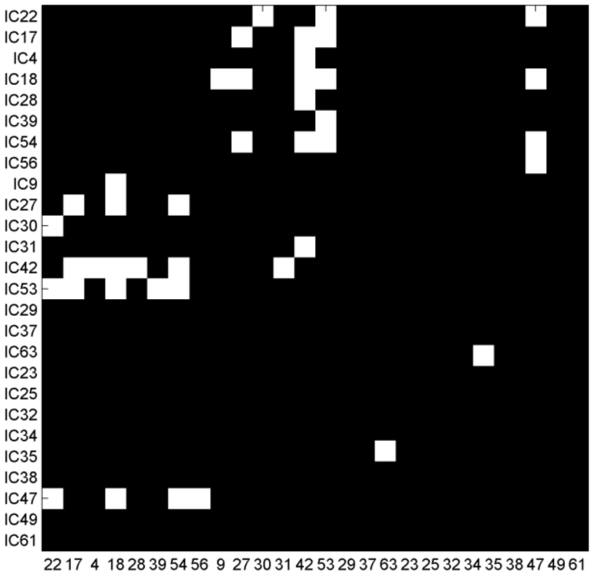

A binary adjacency matrix for the detected significant network. White box indicated connection was significant. The number in the x-axis and y-axis denoted the component's number.

By using the NBS method[6], one significant network was found, and this network contained 21 connections. Then the post-hoc two-sample t tests was applied to determine altered connection in each pair of the three groups.

1 Szewczyk-Krolikowski, K. *et al.* Functional connectivity in the basal ganglia network differentiates PD patients from controls. *Neurology* **83**, 208-214, doi:10.1212/WNL.0000000000000592 (2014).

2 Seeley, W. W. *et al.* Dissociable intrinsic connectivity networks for salience processing and executive control. *J Neurosci* **27**, 2349-2356 (2007).

3 Smith, S. M. *et al.* Correspondence of the brain's functional architecture during activation and rest. *Proc Natl Acad Sci U S A* **106**, 13040-13045, doi:10.1073/pnas.0905267106 (2009).

4 Allen, E. A. *et al.* A baseline for the multivariate comparison of resting-state networks. *Front Syst Neurosci* **5** (2011).

5 Manoliu, A. *et al.* Insular dysfunction within the salience network is associated with severity of symptoms and aberrant inter-network connectivity in major depressive disorder. *Front Hum Neurosci* **7**, 930 (2014).

6 Zalesky, A., Fornito, A. & Bullmore, E. T. Network-based statistic: identifying differences in brain networks. *Neuroimage* **53**, 1197-1207, doi:10.1016/j.neuroimage.2010.06.041 (2010).
